# Significant differences in terms of codon usage bias between bacteriophage early and late genes: a comparative genomics analysis

**DOI:** 10.1186/s12864-017-4248-7

**Published:** 2017-11-13

**Authors:** Oriah Mioduser, Eli Goz, Tamir Tuller

**Affiliations:** 1 Department of Biomedical Engineering, Tel-Aviv University, Ramat Aviv, Israel; 2 SynVaccineLtd. Ramat Hachayal, Tel Aviv, Israel; 3 Sagol School of Neuroscience, Tel-Aviv University, Ramat Aviv, Israel

**Keywords:** Viral evolution, Codon usage bias (CUB), Bacteriophage genome evolution, Viral life cycle, Coding regions, Synthetic virology

## Abstract

**Background:**

Viruses undergo extensive evolutionary selection for efficient replication which effects, among others, their codon distribution. In the current study, we aimed at understanding the way evolution shapes the codon distribution in early vs. late viral genes in terms of their expression during different stages in the viral replication cycle. To this end we analyzed 14 bacteriophages and 11 human viruses with available information about the expression phases of their genes.

**Results:**

We demonstrated evidence of selection for distinct composition of synonymous codons in early and late viral genes in 50% of the analyzed bacteriophages. Among others, this phenomenon may be related to the time specific adaptation of the viral genes to the translation efficiency factors involved at different bacteriophage developmental stages. Specifically, we showed that the differences in codon composition in different temporal gene groups cannot be explained only by phylogenetic proximities between the analyzed bacteriophages, and can be partially explained by differences in the adaptation to the host tRNA pool, nucleotide bias, GC content and more.

In contrast, no difference in temporal regulation of synonymous codon usage was observed in human viruses, possibly because of a stronger selection pressure due to a larger effective population size in bacteriophages and their bacterial hosts.

**Conclusions:**

The codon distribution in large fractions of bacteriophage genomes tend to be different in early and late genes. This phenomenon seems to be related to various aspects of the viral life cycle, and to various intracellular processes. We believe that the reported results should contribute towards better understanding of viral evolution and may promote the development of relevant procedures in synthetic virology.

**Electronic supplementary material:**

The online version of this article (10.1186/s12864-017-4248-7) contains supplementary material, which is available to authorized users.

## Background

Deciphering the regulatory information encoded in the genomes of phages and other viruses, and the relation between the nucleotide composition of the coding regions and the viral fitness is of great interest in recent years.

Gene expression within different Deoxy ribonucleic Acid (DNA) viruses or viruses with DNA intermediate, such as herpeses, lenti-retro, polyoma, papilloma, adeno, parvo and various families of bacteriophages is regulated in a temporal fashion and can be divided into early and late stages with respect to the viral replication cycle [1–8].

The early genes are expressed following the entry into the host cell and code typically for non-structural proteins that are responsible for different regulatory functions in processes such as: viral DNA replication, activation of late genes expression, trans-nuclear transport, interaction with the host cell, induction of the cell’s DNA replication machinery necessary for viral replication, etc. [9, 10]. Late genes largely code for structural proteins required for virion assembly; they are generally highly expressed and their expression is usually induced or regulated by the early genes [9, 10].

Several studies have shown that viral codon frequencies tend to undergo evolutionary pressure for specific CUB; among others, it was suggested that viral CUB is under selection for improving the viral fitness, and in specifically the viral gene expression [11–33].

In particular, in [17] different trends of translation efficiency adaptation of the coding regions of the bacteriophage Lambda early and late genes were demonstrated. Specifically, it was shown that the preferences of codons in early genes, but not in the late genes, were similar to those of the bacterial host [17]. The analysis of ribosome profiling data revealed that the codon decoding rates of viral genes tend to correlate with their expression levels [17]. Interestingly, during the initial stages of phage development the decoding rates in early genes were found to be higher than the decoding rates in late genes; in more progressive viral cycles an opposite trend was demonstrated [17].

In this study we go further, and perform a comparative genomics analysis of the temporal differences in CUB in almost all known viruses with existing in the literature classification of their genes into early and late groups. Specifically basing on analysis of 14 bacteriophages and 11 human viruses we suggest that 50% of the analyzed bacteriophages tend to undergo an extensive evolutionary selection for distinct compositions of synonymous codons in early and late viral genes. We analyze the features of the genomes that undergo this type of selection and argue that the differential CUB can be related to various intracellular phenomena and processes, such as: translational selection and regulation [11, 12, 17, 21, 22, 28, 31], mutational bias and pressure [16, 20, 21, 23, 26, 27, 30, 32, 33], amino acids (AA) compositions [12, 16], and other genomic characteristics, some of which are still not fully understood [13, 14, 29, 34].

Finally, we discuss a possible application of our findings to synthetic virology. Specifically, we suggest using the temporally regulated CUB for controlling the viral gene expression at different time points during the life cycle for designing of optimized and/or deoptimized synthetic viruses which can be used in exploring novel strategies in vaccination (e.g. life attenuated vaccines) and cancer therapy (oncolytic viruses).

## Results

**Fig. 1:**
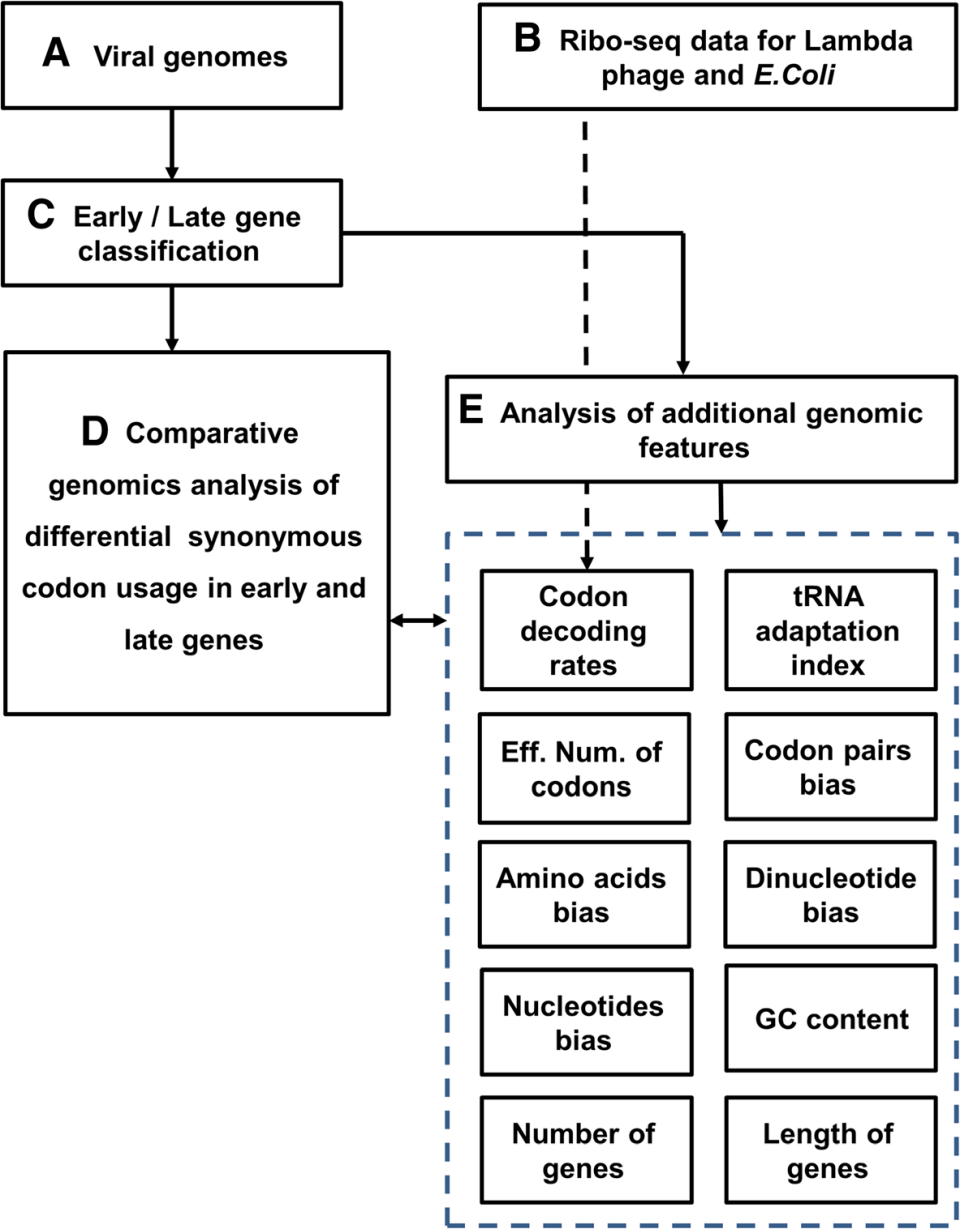
The research outline of the study. The details can be found in the main text: Our analysis was based on coding sequences of 14 bacteriophages and 11 human viruses (**A.**), and on the ribo-seq measurements of bacteriophage Lambda and its *E.coli* host (**B.**). Basing on the existing literature, classification of the viral genes to early and late (with respect to the beginning of the lytic phase) was derived (**C.**). **A.**, **B.**, and **C**, were used to perform a comprehensive comparative genomics analysis of differential synonymous codon usage in early and late genes (**D.**), as well as of additional genomic features possibly related to codon bias (**E.**), such as: ribo-seq based codon typical decoding rates (TDR), Transfer Ribonucleic Acid (tRNA) adaptation indexes (tAI), effective number of codons (ENC), codon pairs bias (CPB), amino acids bias (AAB), dinucleotide bias (DNTB), nucleotide bias (NTB), GC content, number of genes in each temporary group and their length

The research outline of the study is described in Fig. 1. More details can be found in the following sections.

### Bacteriophage early and late genes tend to have different compositions of synonymous codons

Genome level information about the different viruses analyzed in this study, like their hosts, number of genes, gene lengths and ENC, is displayed in Additional file 1: Table S1 and Figure S1.

In order to compare the synonymous codons usage in early and late genes, each coding sequence was represented by its relative synonymous codons frequencies (RSCF) - a 61 dimensional vector expressing each sense codon by its frequency in that sequence normalized relative to the frequencies of other synonymous codons coding for the same AA. We then performed a clustering analysis, assuming that RSCF vectors that are closer with respect to Euclidian metric correspond to genes with a more similar content of synonymous codons (see Materials and Methods).

**Fig. 2:**
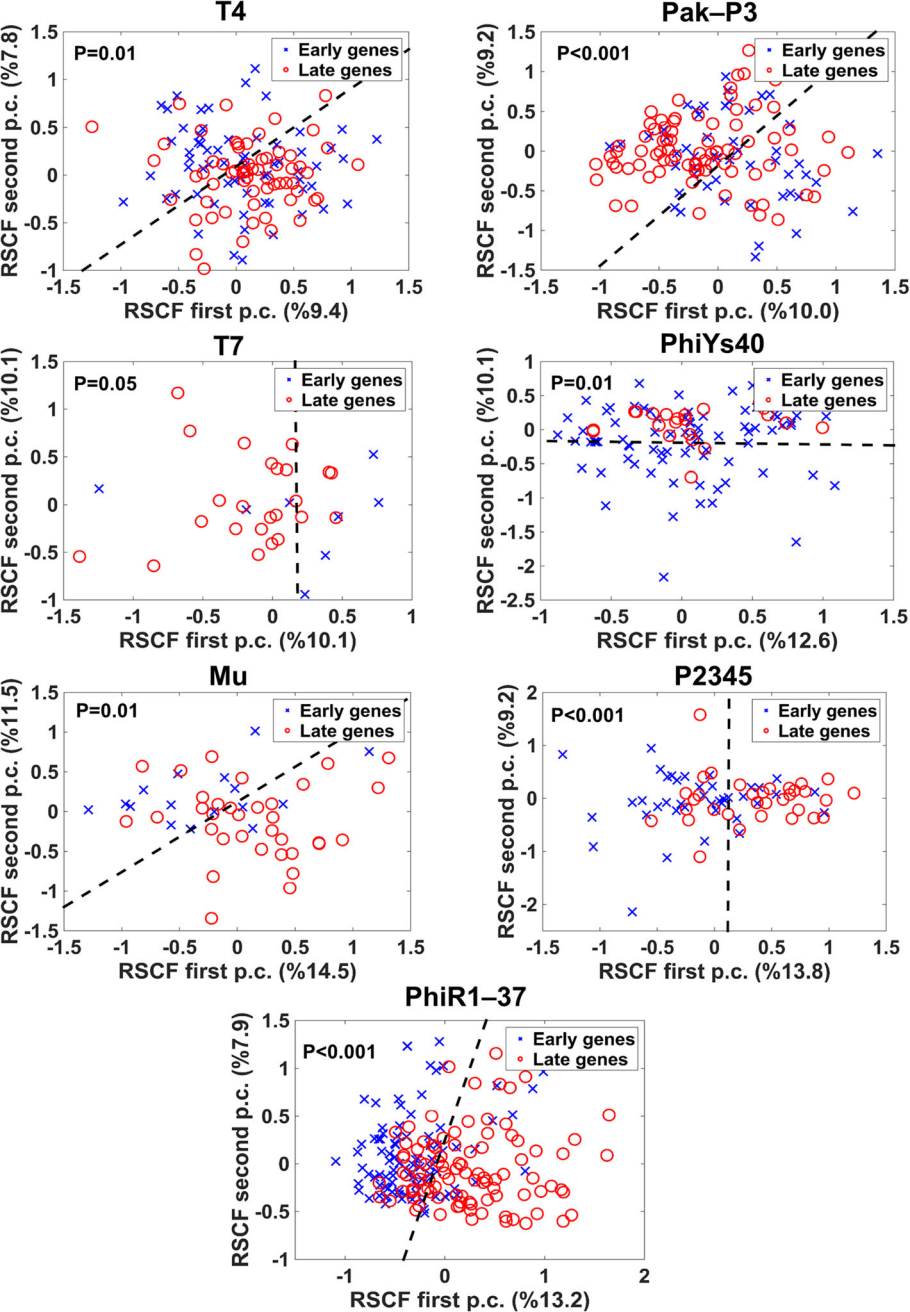
Principal component analysis (PCA) of RSCF vectors for bacteriophages with significant separation in codon usage between early (blue circles) and late (red circles) genes. In order to visualize the clustering, PCA was applied to project the RSCF vectors to a plane spanned by their first two principal components. In order to visualize the separation between clusters a maximum margin separation line, a line for which the Euclidian distance between it and the nearest point from either of the groups is maximized, was calculated and plotted. The significance of cluster separation was assessed by comparing the Davies-Bouldin cluster score to the randomized scores obtained from 100 permutations of gene temporary (early or late) labels. The variances % of the first two principal components are mentioned in the figures axis

**Fig. 3:**
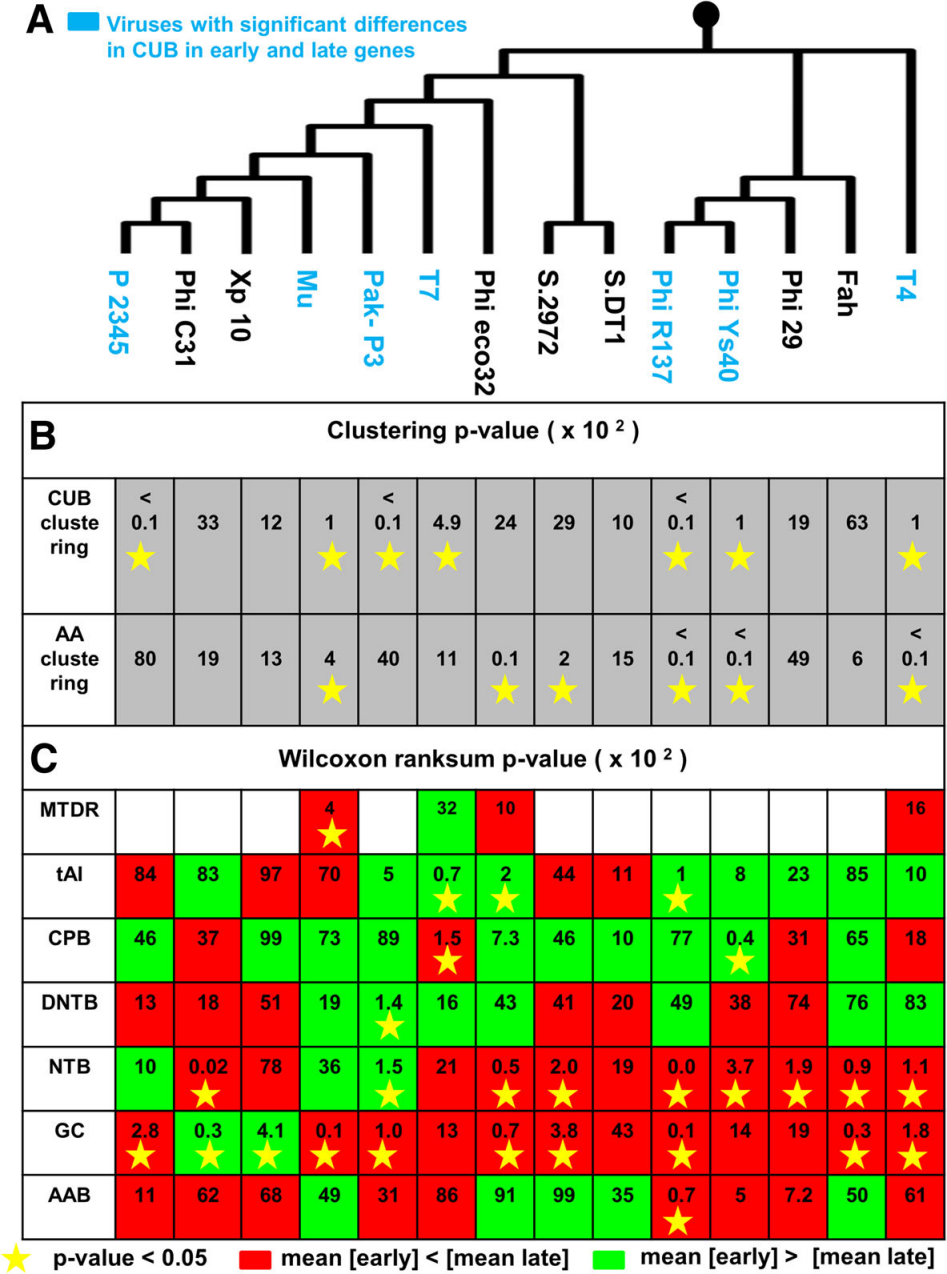
Comparative analysis of early and late genes in 14 different bacteriophages. Details can be found in the main text. **a** A phylogenetic tree built from complete phage proteomes using ARS distance (see Materials and Methods). Phages with significant differences in temporary codon usage are marked by blue. **b** Viruses with significant (*p*-value <0.05) separation between early and late genes w.r.t synonymous codons or AA are marked by yellow stars. **c** Significance of separation between early and late genes w.r.t additional genomic features estimated by Wilcoxon ranksum p-value. Features/viruses with significant (p-value <0.05) separation between the two temporal groups are marked by yellow stars; green is related to higher mean in the case of the early genes and red is related to higher mean in the case of the late genes

Our results suggest that early and late genes in 50% of the analyzed bacteriophages tend to exploit different synonymous codons. Specifically, in 7 of the 14 analyzed bacteriophages, early and late genes were found to be significantly (*p*-value ≤0.05) separated according to the frequencies of their synonymous codons (Figs. 2, 3a, b, Additional file 1: Figure S2 and Figure S3 in Section 1.2). Our analysis provide evidence that different sets of synonymous codons in early vs. late genes are selected for in the course of viral evolution; these differences may be related to the optimization of bacteriophage fitness in different phases of the viral lifecycles.

In addition, 6 out of 14 bacteriophages were also found to be significantly (*p*-value <0.05) separated according to the AA composition of their early and late genes (Fig. 3b, Additional file 1: Figure S4 and Figure S5 in Section 1.2). 4 viruses were characterized both by a differential synonymous codon usage and by a differential AA usage in their early and late genes. These findings suggest that among others, the different codon distribution in early and late genes may be partially related to the functionality of the encoded proteins via their AA content and possibly protein folding [35].

To check if bacteriophages with significant differences in synonymous codons usage in temporal genes tend to have more similar genomic sequences (usually related to smaller evolutionary distances), we reconstructed a phylogenetic tree of the bacteriophage proteomes based on Average Repetitive Subsequences (ARS) distance matrix and neighbor joining method as described in Materials and Methods section and in references therein (Fig. 3a). We then performed a statistical analysis in order to investigate the relation between the differences in temporal regulation of synonymous codons in different viruses and their evolutionary distances. We did not find such a relation (see details in Additional file 1: Section 1.3 and Figure S6), suggesting that the differential codon usage in early and late genes is a complex trait related to alternative determinants such as the bacterial niche, the specific phage proteins and their function/structure, etc.

Viruses undergo an extensive evolutionary selection for adaptation to their host’s cell environment, and thus it can be assumed that their codon composition reflects an efficient adaptation of the viral genes to specific intracellular conditions (e.g. in terms of gene expression factors such as tRNA molecules, AA concentration, etc) that are prevalent in different gene expression stages, in accordance with the reported results.

### Weaker separation between synonymous codon usage in early and late genes in human viruses

The results in the previous section suggest that bacteriophages undergo an extensive evolutionary selection on a synonymous level for temporal regulation of gene expression. Whether this also occurs in viruses of humans and other eukaryotic hosts is harder to ascertain. Human Immunodeficiency Virus 1 (HIV-1) was found to have a significant separation (*p*-value ≤0.05) of codon composition between early and late genes, while such separation was not statistically significant in the rest of the analyzed viruses (see Additional file 1: Table S2 in Section 1.4).

As evidenced in Additional file 1: Table S1 and Figure S1, human viruses tend to have fewer genes than bacteriophages. Therefore, we were interested in checking whether this fact can explain the weaker signal for temporal separation in CUB, and if, in practice, human viruses may also behave as bacteriophages with respect to the differential usage of synonymous codons in their early and late genes. To this end we analyzed the 7 bacteriophages with temporary differential codon usage by sampling in each one of them a number of early and late genes that is typical to human viruses (average of 8 early genes and 14 late genes). We found that the temporal differences in codon usage remained significant even after randomly reducing the number of genes, indicating, among others, that these differences cannot be directly explained only by the genome size.

### Comparison of early and late genes with respect to additional features of their coding regions

The signal of selection for temporarily regulated composition of synonymous codons in bacteriophages demonstrated in the previous subsection led us to analyze additional genomic features, such as: codon mean typical decoding rate (MTDR), tRNA adaptation index (tAI), codon pairs bias (CPB), dinucleotide bias (DNTB), nucleotide bias (NTB), GC content and amino acids bias (AAB).

Various studies related these features to different genomic mechanisms and biological processes involved in viral replication cycles and are related to the viral fitness.

For example, it was suggested that gene translation efficiency can be affected not only by single codons, but also by distribution of codon pairs [36]. In [37–39] it was argued that pairs of adjacent nucleotides may be an important genomic characteristic being under a significant evolutionary pressure in viruses and their hosts; specifically, it was suggested that CpG pairs are under-represented in many Ribonucleic Acid (RNA) and in most small human DNA viruses, in correspondence to dinucleotide frequencies of their hosts. This phenomenon can be related, for example, to the contribution of the CpG stacking basepairs to RNA folding [40] and/or to the enhanced innate immune responses to viruses with elevated CpG [41]. The stability of the RNA secondary structures can be also affected by the genomic composition of nucleotides and in particular by GC content [42]. In addition, nucleotide compositions and AA usage bias may affect, among others, the synthesis of viral molecules, and the function and structure of the encoded proteins.

Consequently, we estimated the listed above features for all genes in all viruses, and evaluated the separation between early and late genes with respect to each one of them (see Materials and Methods). The results shown in Fig. 3c suggest that the differential usage of synonymous codons in early and late genes can be partially related to temporal differences in various characteristics of genomic sequences. Specifically, the features with the strongest temporal differences are the NTB and GC content which are significant (*p*-value <0.05) in most of the phages.

In addition, we wanted to check if the bacteriophages with a significant temporal separation with respect to synonymous codons tend also to be enriched with specific genomic features in comparison to the group of bacteriophages with non-significant temporal differences in synonymous codons. To this end, we compared the distribution of various genomic features in the two groups. Based on Wilcoxon ranksum test we found no significant differences between the two groups of bacteriophages in terms of: genome length (*p*-value = 0.53), ENC (p-value = 0.4), CPB (p-value = 0.99), DNTB (p-value = 0.21), NTB (p-value = 0.9), GC content (p-value = 0.8) and AAB (p-value = 0.99). See also Additional file 1: Figure S7 in Section 1.5.

## Discussion

In this study, we performed a comparative genomics analysis of viruses with annotations in literature regarding their genes division according to temporal expression. We examined 14 bacteriophages with different bacterial hosts and 11 human viruses in order to understand if there is a universal difference in synonymous codons usage as well as in additional genomic features (such as codon decoding rates, nucleotide/dinucleotide/AA biases, GC content and others) with respect to different temporal stages of viral life cycle.

Our results suggest that 50% of bacteriophages undergo an extensive evolutionary selection for distinct compositions of synonymous codons in early and late viral genes. This phenomenon was found to be weaker/less significant in human viruses, possibly because of the stronger selection pressure in bacteriophages / bacteria due to the larger size of their populations, and because of the fact that regulation processes in human gene expression are more ‘complex’ and thus may be mediated by additional aspects not necessary related to codons.

The differences between early and late genes, both with respect to the composition of synonymous codons and with respect to additional genomic features described in the previous sections, can be possibly influenced by various intracellular phenomena and processes related to the optimization of gene expression and to the overall fitness of the phage. To mention a few, these phenomena/processes include: adaptation of translation elongation efficiency in different phases of the viral lifecycle [17], Messenger Ribonucleic Acid (mRNA) folding [43, 44], adaptation of the viral genes to the (possibly altering) tRNA pool of their hosts [11, 12, 17, 31], mutation levels and biases [16, 20, 21, 23, 26, 27, 30, 32, 33], transcription regulation [45, 46], protein function and structure [47], cell metabolism [48], etc.

There can be various explanations to the fact that it seems that only 50% of the bacteriophages there is a significant difference in the codon usage in early vs. late genes:

First, it is possible that the effective population size (which is not easy to estimate) varies among the analyzed bacteriophages. The selection pressure is weaker in bacteriophages with smaller population size.

Second, this observation may be also related to the intracellular regimes during the development of the different bacteriophages. For example, it is possible that during the development of some bacteriophages the tRNA levels are modulated/changed, while in other cases the changes are minor. The changes in the tRNA levels may trigger evolution of different CUB in early/late genes in the bacteriophages that experience them.

Third, this result may be related to the nature of the protein encoded in the bacteriophages genome. The specific function and properties of the proteins in different bacteriophages may affect the observed levels of selection. For example, it is possible that only in some bacteriophages the early vs. late genes tend to have different structure with different co-translational folding constraints that eventually affect the codon bias. It is also possible that only in some bacteriophages the early vs. late genes tend to have different expression levels/patterns that eventually affect their codon bias.

It is possible that the results reported here have relevant practical applications. For example, vaccines, and their discovery, are topics of singular importance in present-day biomedical science; however, the discovery of vaccines has hitherto been primarily empirical in nature requiring considerable investments of time, efforts and resourced. To overcome the numerous pitfalls attributed to the classical vaccine design strategies, more efficient and robust rational approaches based on computer-based methods are highly desirable. One direction in designing in-silico vaccine candidates may be based on exploiting the temporally regulated synonymous information encoded in the genomes and investigated in this study for attenuating the viral replication cycle while retaining the wild type proteins. In particular, the result reported here suggest that viral genes can be designed with respect to phase specific temporary regulated gene expression constraints, and this design would result in controllable yields of the corresponding genetic products during a defined time period. To achieve this, codons would be selected with frequencies maximally dissimilar / similar to the set of early or late genes than a random set of genes. See Additional file 1: Section 2 and Figures S8, S9 for more details and examples.

## Conclusions

The codon distribution in large fractions of bacteriophage genomes tend to be different in early and late genes. It seems that various additional genomic features (e.g. NTB and GC content) tend to be associated with this signal. This phenomenon seems to be related to various aspects of the viral life cycle, and to various intracellular processes. A similar signal may be observed in human viruses but it seems significantly less frequent. We believe that the reported results should contribute towards better understanding of viral evolution and may promote the development of relevant procedures in synthetic virology.

## Material and methods

The research outline of the study is described in Fig. 1.

### Viruses

Human Viruses analyzed in this study include Herpes viruses, papilloma viruses, Polyomavirus and HIV.

The analyzed bacteriophages include: bacteriophage Lambda, bacteriophage T4, bacteriophage Pak P3, bacteriophage phi29, bacteriophage T7, bacteriophage phiYs40, bacteriophage Fah, bacteriophage xp10, bacteriophage Streptococcus DT1, bacteriophage Streptococcus 2972, bacteriophage Mu, bacteriophage phiC31, bacteriophage phiEco32, bacteriophage p23–45 and bacteriophage phiR1–37.

These viruses were chosen since they have a known division to early and late genes annotated in the literature, as described in Additional file 1: Table S3.

### Synonymous codon usage analysis

Codon composition of a coding sequence was represented by a 61-dimensional vector of RSCF of each one of 61 coding codons (stop codons are excluded).

Clustering analysis was performed on RSCF vectors of each viral coding sequence. Each viral sequence was assigned a group label corresponding to its temporal expression stage (early/late) (according to the classification known in the literature). The tendency of sequences to cluster according to the codons usage in two different clusters corresponding to their temporal expression stages (early/late) was measured using the Davies-Bouldin score (DBS) [49]. This score is based on a ratio of within-cluster and between-cluster distances. The optimal clustering solution has the smallest DBS value.

The significance of cluster separation was assessed by comparing the DBS of the wildtype sequences to the randomized scores obtained from 1000 permutations of gene group labels (early or late).

In addition, a similar analysis was performed on AA frequencies as well.

More details can be found in Additional file 1: Section 3.3.

We decided to use the RSCF, since in this study we are interested in comparing the frequencies of the codons without an a-priory assumption/focus on relative bias of codons; to this aim it is more natural to use the RSCF rather the widely used Relative Synonymous Codons Usage (RSCU) measure [50]. However, these measures are similar, and the same analysis performed with RSCU does not change the final conclusions.

### Additional genomic features analyzed in this study

**The tRNA adaptation index (tAI)** quantifies the adaptation of a coding region to the tRNA pool with parameters describing the different tRNAs copy numbers and the selective constraints on the codon–anti-codon coupling efficiency. Since, currently, these parameters are based on gene expression measurements in a very limited number of organisms, and since the efficiencies of the different codon-tRNA interactions are expected to vary among different species, we used a novel approach proposed in [51] for adjusting the tAI weights to any target organism, without the need for gene expression measurements, basing on an optimization of the correlation between the tAI and a measure of codon usage bias. It is the first time, to our knowledge, that this approach is applied to study tAI in viruses with respect to their hosts. The resulting tAI values were computed by a standalone application [52]. See more details in Additional file 1: Section 3.4.

**Effective number of codons (ENC)** is a measure that quantifies how far the synonymous codon usage of a gene departs from what is expected under the assumption of uniformity [53]. See more details in Additional file 1: Section 3.5.

**GC-content** is the percentage of nitrogenous bases on a DNA or RNA molecule that are either guanine or cytosine. See more details in Additional file 1: Section 3.6.

**Codon pair bias (CPB).** To quantify the CPB, we follow [54] and define a codon pair score (CPS) as the log ratio of the observed over the expected number of occurrences of this codon pair in the coding sequence. The CPB of a virus is then defined as an average CPSs over all codon pairs comprising all viral coding sequences. See more details in Additional file 1: Section 3.7.

**Dinucleotide bias (DNTB).** We define a dinucleotide score (DNTS) for a pair of nucleotides as an observed over expected ratio of its occurrences in a sequence. The DNTB of a virus is defined as an average of DNTSs over all dinucleotides comprising all viral coding sequences. See more details in Additional file 1: Section 3.8.

**Nucleotide (NTB) and amino acid (AAB) biases** are defined as a normalized Shannon entropy over the frequencies of the nucleotides / AA in a genomic sequence. See more details in Additional file 1: Section 3.9.

### Ribosome profiling analysis

**Ribosome profiling (ribo-seq) data** was taken from [55]. Ribosome profiles for bacteriophage Lambda and *Escherichia coli* (*E.Coli)* were reconstructed and normalized as in [17]. The normalization enables measuring the relative time a ribosome spends translating each codon in a specific gene relative to other codons, while considering the total number of codons in this gene, and results in codons normalized footprint count (NFC).

**Codon typical decoding rate (TDR).** Following [17], in order to estimate the typical decoding time of each codon based on the corresponding ribo-seq data, we used a novel statistical model [56] which takes into consideration the skewed nature of the NFC distribution and describes the NFC histogram of each codon as an output of a random variable which is a sum of a normally distributed and an exponentially distributed random variables called Exponentially Modified Gaussian (EMG). Maximum likelihood criterion was used to estimate the parameters of these distributions for each codon according to the ribo-seq data by fitting the suggested model to the NFC distribution. The mean of the normal distribution component of EMG was called $\mu, \mathrm{and}\ \frac{1}{\upmu}$ was defined to be the TDR of a codon [17]. See more details in Additional file 1: Section 3.10.

**Mean typical decoding rate (MTDR)** is a measure which estimates the global translation elongation efficiency of the entire gene as a geometric average of TDRs of its codons. See more details in Additional file 1: Section 3.11.

Since bacteriophage Lambda is the only phage with publicly available ribo-seq data, a direct analysis of TDRs of other phages is currently impossible. Nevertheless, due to the adaptation of the viruses to the translation machinery of their hosts, a rough estimation of MTDR values for other *E.Coli* phages rather than Lambda may be obtained from the available ribose-seq of the *host* genes.

### Phylogenetic reconstruction

Following [57], a phylogenetic reconstruction of bacteriophages was performed basing on an alignment-free distance that estimates the similarity of two sequences (in our case entire viral proteomes) according to the average length of subsequences that are repeated in both of them (the ARS). The tree was built using the neighbor joining algorithm as implemented in [58].

See more details in Additional file 1: Section 3.12.

## Additional files

Additional file 1: Supplementary results and material. (PDF 2568 kb)

Additional file 2: Full list of the analyzed viruses including their accession numbers and temporal labels of genes. (XLSX 111 kb)

## Abbreviations

AA: Amino acid
AAB: Amino acid bias
ARS: Average repetitive subsequences
CPB: Codon pair bias
CPS: Codon pair score
CUB: Codon usage bias
DBS: Davies-bouldin score
DNA: Deoxy ribonucleic acid
DNTB: Dinucleotide bias
DNTS: Dinucleotide score
E.Coli: *Escherichia coli*
EMG: Exponentially modified gaussian
ENC: Effective number of codons
HIV: Human immunodeficiency virus
mRNA: Messenger ribonucleic acid
MTDR: Mean typical decoding rate
NFC: Normalized footprint count
NTB: Nucleotide bias
PCA: Principal component analysis
RNA: Ribonucleic acid
RSCF: Relative synonymous codons frequencies
RSCU: Relative synonymous codons usage
tAI: tRNA adaptation index
TDR: Typical decoding rate
tRNA: Transfer ribonucleic acid
