# Supplementary material for: Significant differences in terms of codon usage bias between bacteriophage early and late genes: a comparative genomics analysis
Source: BMC Genomics. 2017 Nov 13;18:866. doi: 10.1186/s12864-017-4248-7 (PMC5683454; doi:10.1186/s12864-017-4248-7)
Supplement: Supplementary file 1 — Supplementary results and material. (PDF 2568 kb) [file 12864_2017_4248_MOESM1_ESM.pdf]

# Supplementary material: Significant differences in terms of codon usage bias between bacteriophage early and late genes: A comparative genomics analysis

Oriah Mioduser[1,&], Eli Goz[1,2,&], Tamir Tuller[1,2,3,*]

1 Department of Biomedical Engineering, Tel-Aviv University, Ramat Aviv, Israel.
2 SynVaccineLtd. Ramat Hachayal, Tel Aviv, Israel. 3 Sagol School of Neuroscience, Tel-Aviv University, Ramat Aviv, Israel.

&Equal contribution. *Corresponding author (TT): tamirtul@post.tau.ac.il

## 1. Results

### 1.1. Genomic properties of different viruses and temporal classification of their genes

**Table S1.** *Genomic properties of different viruses*

| **Virus** | **Host** | **Number of genes** | | **Average genes length** | | **Average ENC** | |
|---|---|---|---|---|---|---|---|
| | | **Early** | **Late** | **Early** | **Late** | **Early** | **Late** |
| **Phages** | | | | | | | |
| **T4** | E.coli | 64 | 64 | 589.4 | 1009.0 | 34.37 | 37.36 |
| **Pak_P3** | Pseudomonas aeruginosa | 51 | 83 | 311.8 | 641.6 | 35.25 | 38.20 |
| **phi29** | Bacillus subtilis | 16 | 11 | 396.4 | 1084.6 | 35.92 | 44.16 |
| **T7** | E.coli | 10 | 24 | 635.1 | 905.6 | 35.74 | 39.84 |
| **phiYs40** | Thermus thermophilus | 79 | 22 | 602.8 | 1623.3 | 35.92 | 37.21 |
| **Fah** | Bacillus cereus | 16 | 32 | 679.1 | 707.5 | 36.36 | 35.38 |
| **xp10** | Xanthomonas oryzae | 27 | 24 | 605.7 | 880.0 | 40.61 | 42.01 |

| | | | | | | | |
|---|---|---|---|---|---|---|---|
| **Streptococcus DT1** | Streptococcus thermophilus | 19 | 14 | 519.6 | 1038.2 | 37.90 | 37.15 |
| **Streptococcus 2972** | Streptococcus thermophilus | 16 | 17 | 528.8 | 1009.8 | 37.28 | 38.05 |
| **Mu** | E.coli | 16 | 34 | 492.6 | 767.6 | 38.05 | 39.50 |
| **phiC31** | Streptomyces coelicolor | 27 | 22 | 552.1 | 820.0 | 33.03 | 36.08 |
| **phiEco32** | E.coli | 45 | 27 | 363.9 | 1108.4 | 34.85 | 38.81 |
| **p23-45** | Thermus thermophilus | 40 | 33 | 464.6 | 1196.6 | 35.76 | 37.61 |
| **phiR1-37** | Yersinia enterocolitica | 92 | 97 | 284.3 | 825.3 | 31.38 | 34.80 |
| **Human viruses** | | | | | | | |
| **Simplex virus** | Human | 13 | 40 | 2143.2 | 1641.5 | 39.00 | 38.23 |
| **Varicello virus** | | 13 | 39 | 1989.5 | 1632.3 | 51.04 | 49.25 |
| **Cytomegalo virus** | | 18 | 32 | 1735.8 | 1657.7 | 41.00 | 41.96 |
| **Roseolo virus** | | 12 | 24 | 1914.0 | 1599.4 | 49.79 | 47.60 |
| **Alphapapilloma virus** | | 6 | 2 | 673.0 | 1470.0 | 37.04 | 40.62 |
| **Betapapilloma virus** | | 6 | 2 | 899.5 | 1554.0 | 39.26 | 44.44 |
| **Gammapapilloma virus** | | 5 | 2 | 856.2 | 1558.5 | 39.63 | 42.64 |
| **Mupapilloma virus** | | 5 | 2 | 779.4 | 1525.5 | 39.08 | 45.21 |
| **Nupapilloma virus** | | 6 | 2 | 728.0 | 1708.5 | 42.93 | 49.96 |
| **Polyoma virus** | | 3 | 4 | 908.0 | 762.0 | 35.68 | 38.52 |
| **HIV-1** | | 3 | 6 | 411.0 | 1583.5 | 40.06 | 38.76 |

The full list of the analyzed viruses including their accession numbers and temporal labels of genes can be found in the supplementary file **list_of_viruses.xlsx.**

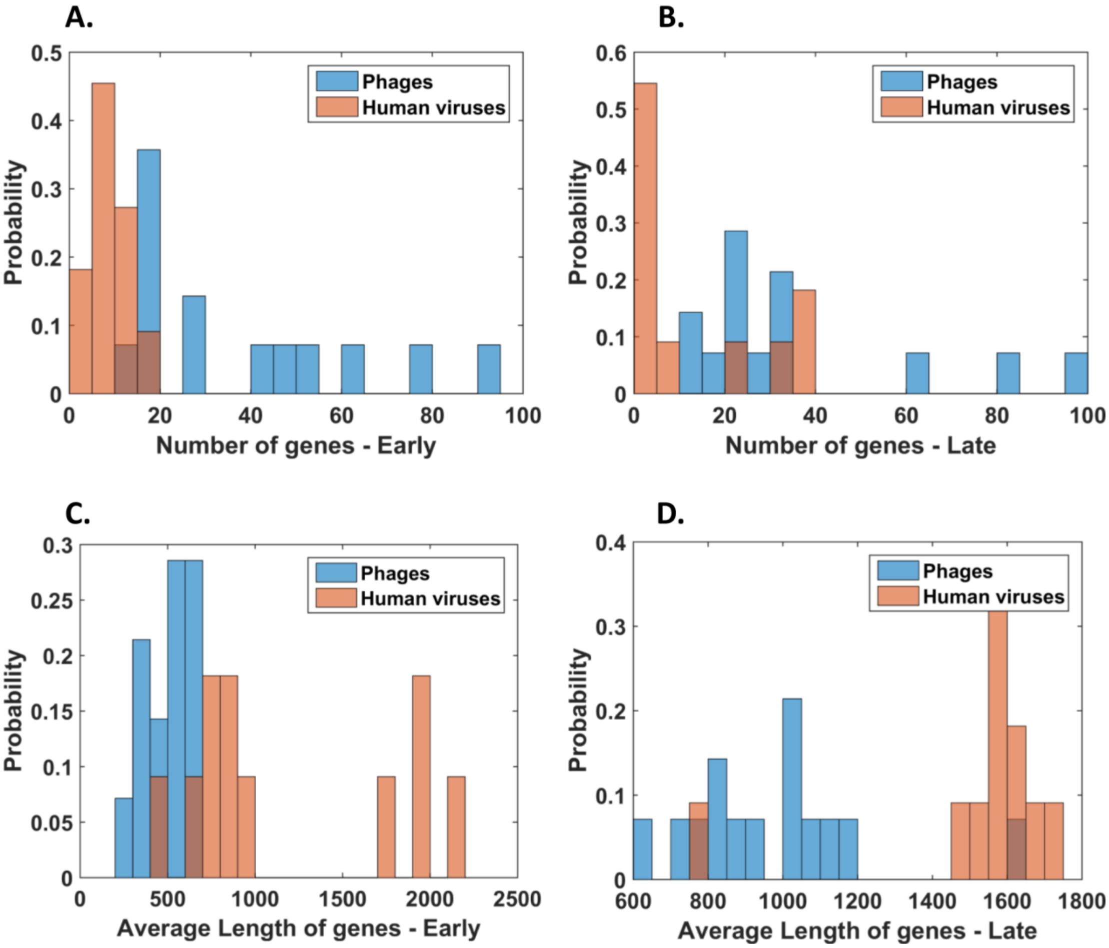


**Figure S1.** *Comparison of number of genes and their lengths in bacteriophages and human viruses*

### 1.2. Differential codon usage bias and amino acids analysis in temporal genes of bacteriophages

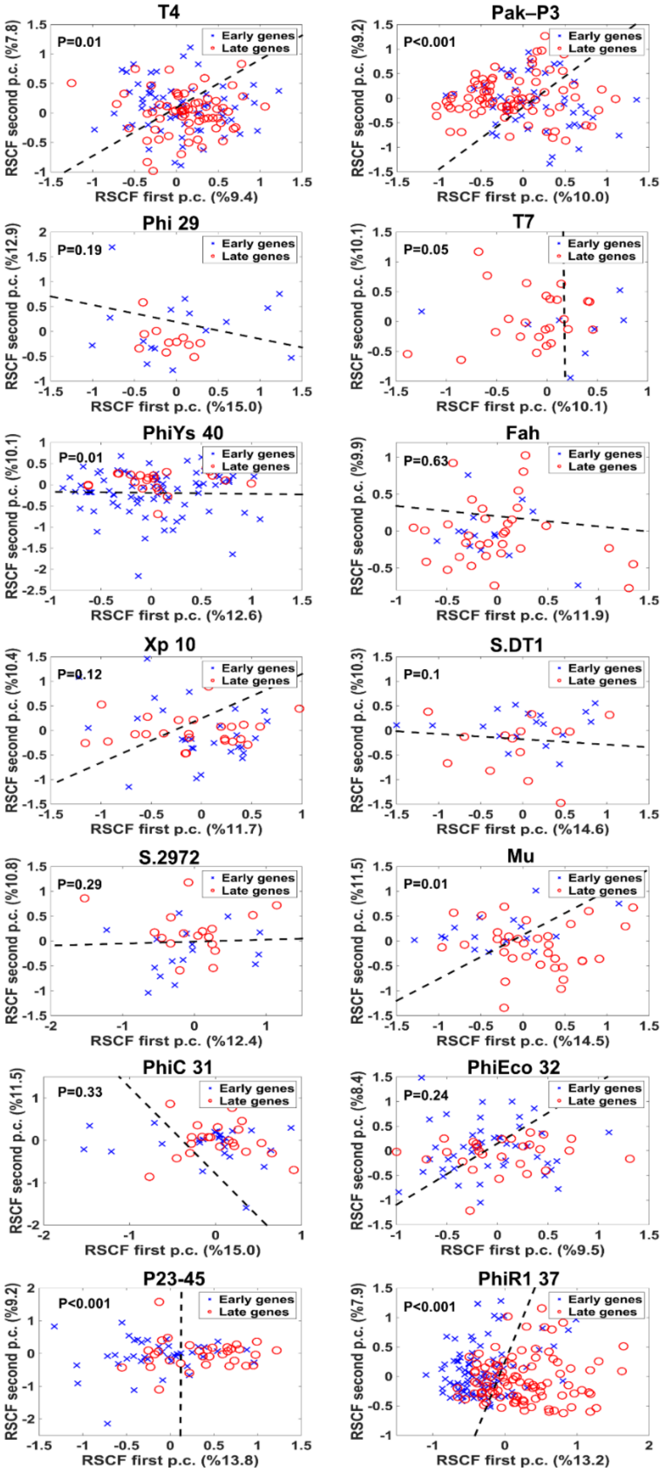


**Figures S2.** *Principal component analysis (PCA) of codon usage for 14 analyzed bacteriophages.*

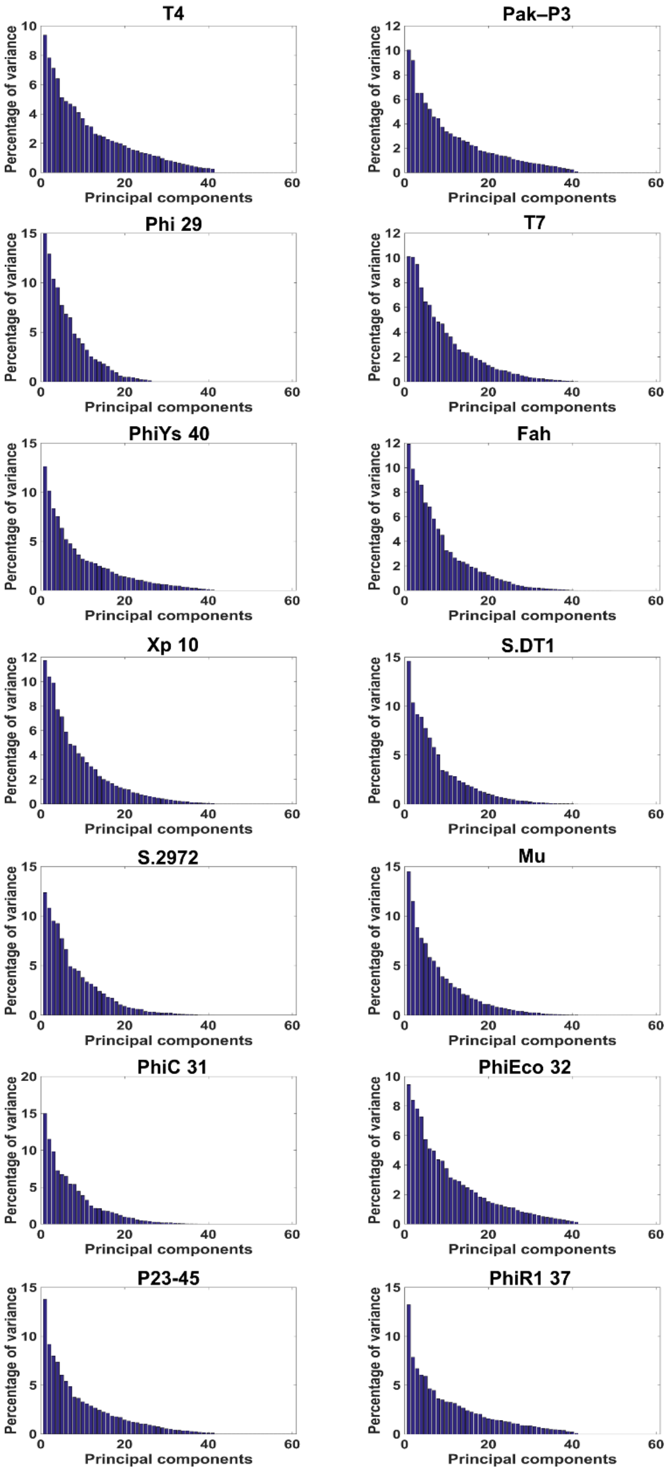


**Figures S3.** *Principal component analysis (PCA) of codon usage variance distribution for 14 analyzed bacteriophages.*

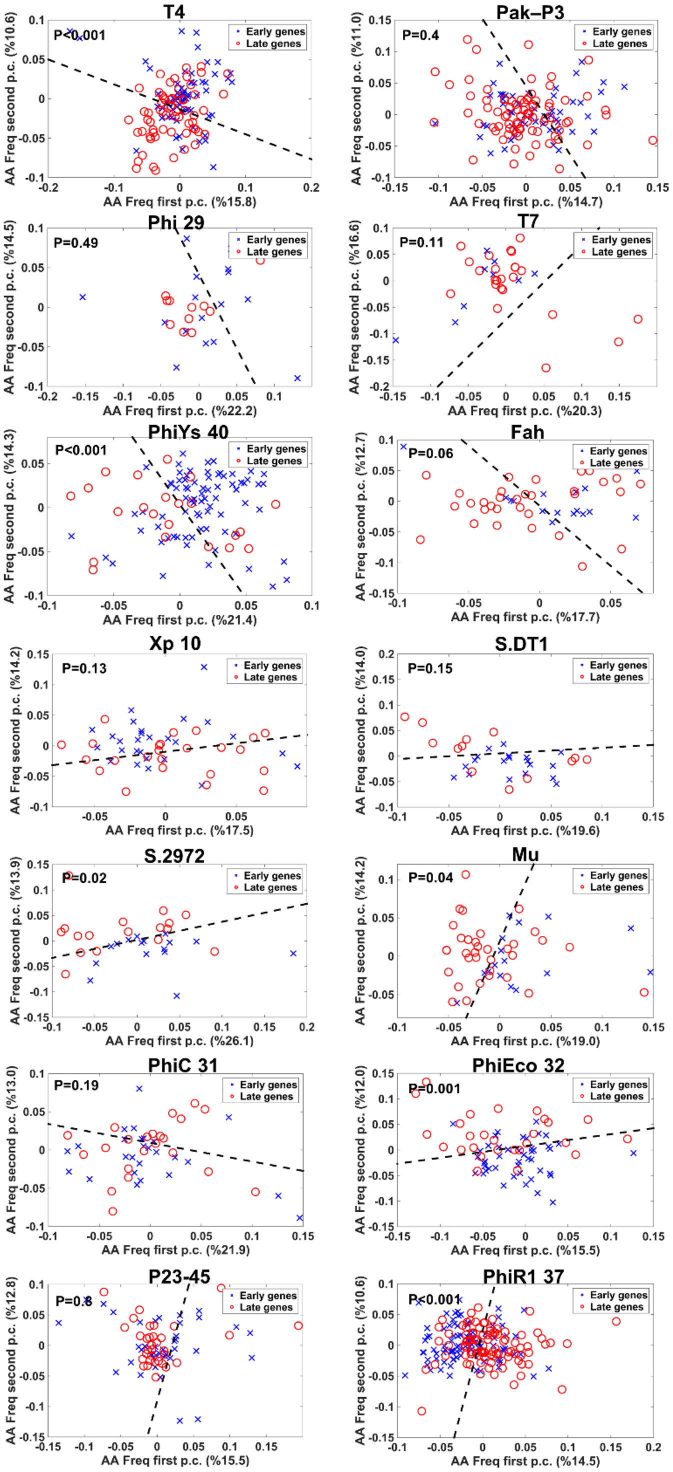


**Figures S4.** *Principal component analysis (PCA) of amino acids usage for 14 analyzed bacteriophages.*

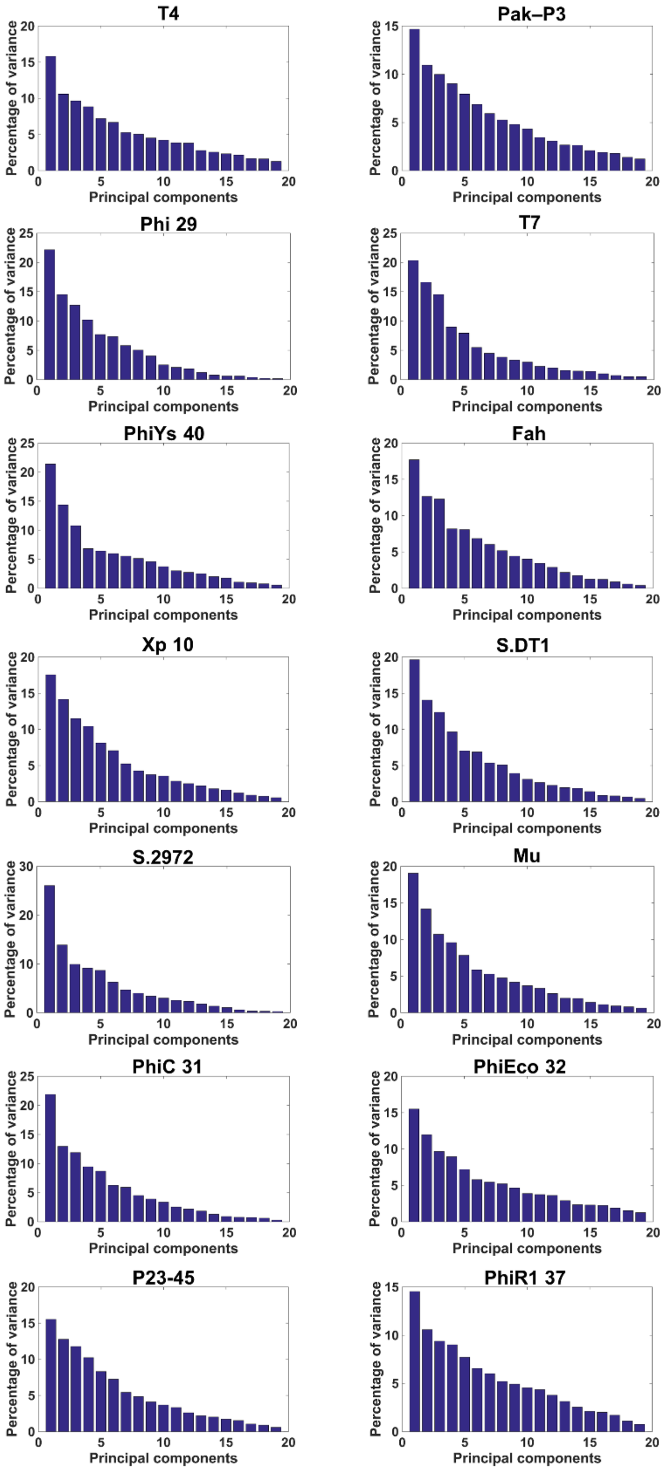


**Figures S5.** *Principal component analysis (PCA) of amino acids usage variance distribution for 14 analyzed bacteriophages.*

### 1.3. Bacteriophages with significant differences in synonymous codons usage in temporal genes do not tend to have more similar genomic sequences than the others

As explained in the main text, we used an alignment free distance measure that estimates the similarity of two sequences (in our case entire viral proteomes) according to the average length of subsequences that are repeated in both of them (average repetitive subsequences; see also section 2.10 bellow and [1]). In order to compare the distances between phages with the signal of temporary differential codon usage to the rest of the phages, we compared the mean distance between all pairs of phages with the signal to the distribution of mean pairwise distances in 100 randomly sampled groups of 7 viruses (the number of viruses in each sample is equal to the number of viruses in the test group). No significant differences in the test group as compared to the randomly sampled groups with respect to genomic similarity was found (empiric p-value = 0.55).

Due to the relatively high similarity between two types of streptococcus (DT1 and 2972), the distribution of mean pairwise distance in the randomly sampled groups was found to be bi-modal (**Figure S6.A**). Repeating a similar analysis after excluding one of the streptococcus, we obtained a uni-modal distribution of random mean pair-distances; again, no significant differences in the test group as compared to the randomly sampled groups with respect to genomic similarity was found (empiric p-value = 0.22, Figure **S6.B**).

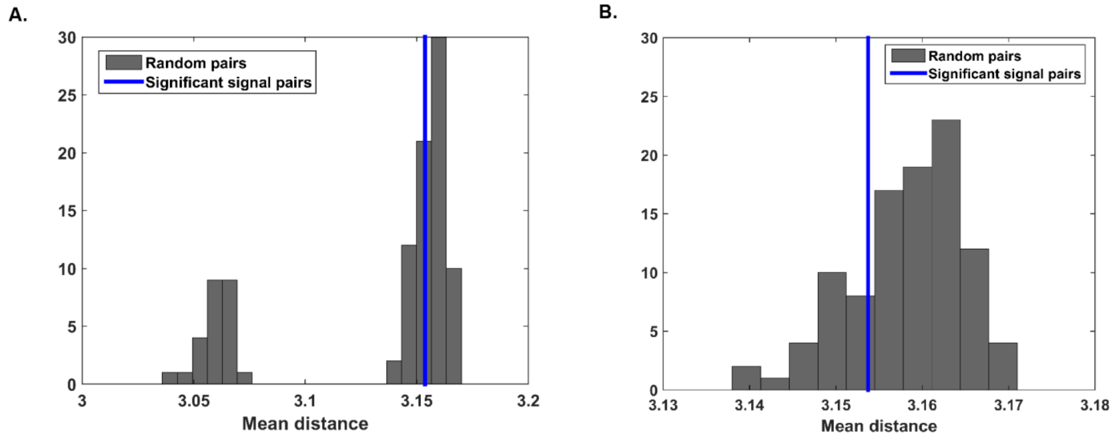


**Figure S6.** *Comparison of a mean pairwise distance between the group of phages with a differential codon usage in temporal genes (blue) to 100 groups of randomly sampled phages of the same size (grey).* ***A.*** *All 14 phages.* ***B.*** *All phages, excluding one streptococcus.*

### 1.4. Analysis of differential codon usage bias (CUB) and amino acid bias (AAB) in temporal genes of human viruses

**Table S2. Weak evidence of differential synonymous codon usage bias in early and late genes in human viruses.** *Entries with significant (p-value $< 0.05$) separation signals are marked by yellow.*

| **Virus** | **Early-late separation according to CUB** | **p-value** | **Early-late separation according to AAB** | **p-value** |
|---|---|---|---|---|
| Simplex virus | No | 0.25 | No | 0.19 |
| Varicello virus | No | 0.85 | **Yes** | **0.02** |
| Cytomegalo virus | No | 0.35 | **Yes** | **0.03** |
| Roseolo virus | No | 0.37 | No | 0.68 |
| Alphapapilloma virus | No | 0.08 | No | 0.17 |
| Betapapilloma virus | No | 0.18 | No | 0.9 |
| Gammapapilloma virus | No | 0.14 | No | 0.09 |
| Mupapilloma virus | No | 0.29 | No | 0.29 |
| Nupapilloma virus | No | 0.29 | No | 0.5 |
| Polyoma virus | No | 0.45 | No | 0.7 |
| HIV-1 | **Yes** | **0.04** | No | 0.21 |

**1.5. Bacteriophages with a significant temporal separation with respect to synonymous codons do not tend to be enriched with specific genomic features in comparison to the group of bacteriophages with non-significant temporal differences in synonymous codons.**

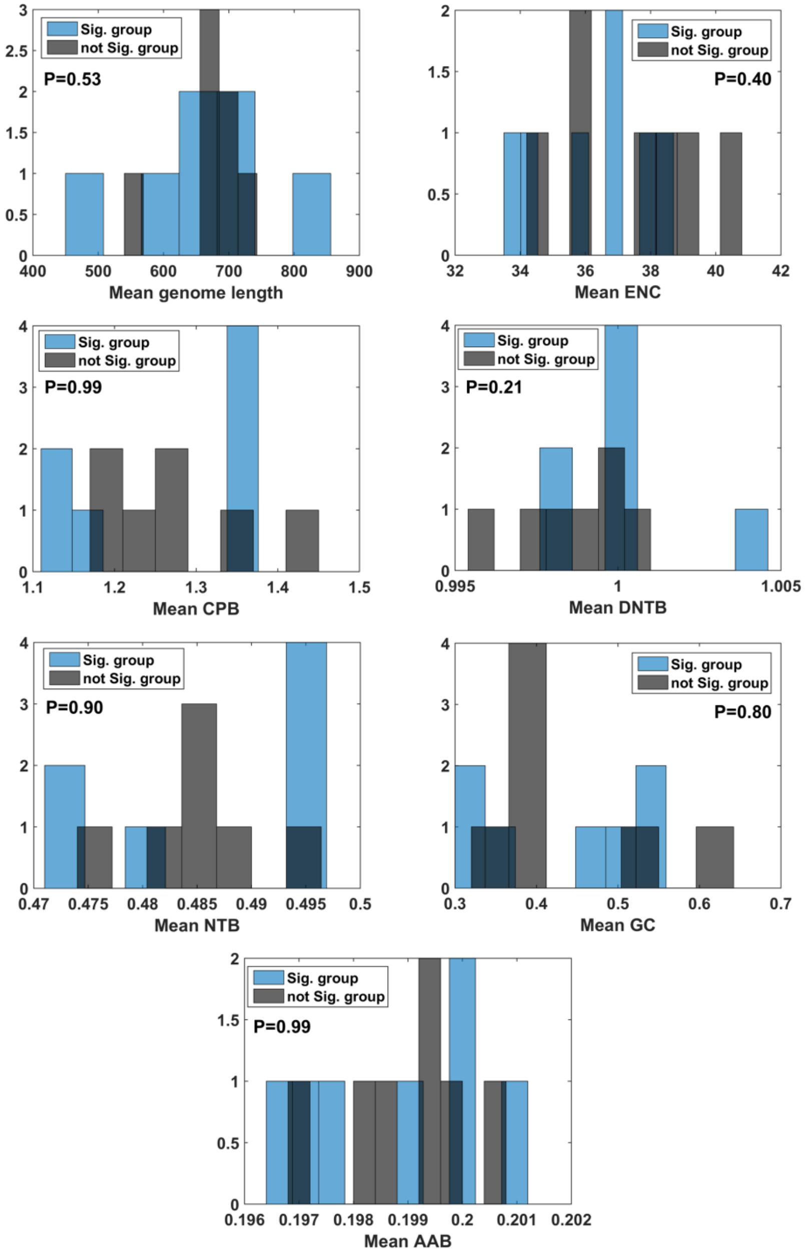


**Figure S7.** *Comparison of different genomic features in viruses with significant temporal separation in codon usage bias (blue) vs. viruses with no such separation (grey). Y axis –*

*probability; X – genomic mean feature values (each virus is represented by an average of feature values over all its genes). Wilcoxon ranksum p-values are specified.*

## 2. Application to synthetic virology

Basing on our findings, we propose a novel method to reengineer viral coding sequences which doesn't require the knowledge of the host's codon usage bias (which can be unknown, tissue and/or cell specific). Instead we suggest engineering genomic sequences, which can be either endogenous viral genes or heterologous transgenes, with respect to a subset of viral genes, clustered according their expression stage (early or later in the replication cycle). Since viruses undergo an extensive evolutionary selection for adaptation to the changing cell environment, it is plausible to assume that their codons composition reflects an efficient modus operandi of the viral machinery at specific replication stage and therefore may be used as a reference set for optimization / de-optimization of synonymous codons. Our findings can be used for controlling the viral gene expression at different time points during the life cycle in two directions: both optimizing it (e.g. for enhancing gene therapies, viral vectors, expressing oncolytic viruses) or de-optimizing it (life attenuated vaccines).

### 2.1. Exploiting temporary differences in synonymous information for de-optimization / optimization of viral genes

Viral genes de-optimization/ optimization can generate efficient live attenuated based vaccines / oncolytic viruses. We suggest performing optimization/ deoptimization of synonymous codons with respect to different temporary stages in the viral life cycle and with no dependence on the cell type, tissue, host, knowledge about highly expressed genes etc.

To achieve this, the following algorithm can be employed:

**Input:**

- Target sequence *t*: a wild-type endogenous viral coding sequences or a coding sequence originating in some heterologous transgene.
- A set of reference sequences *R*: viral coding sequences grouped by expression period (early/late/intermediate).

**Output:**

- A reengineered variant s of the target sequence *t* such that *s* and *t* code for the same protein and the codons fit to one of the stages: early/late/intermediate.
- For each sequence *k* in the reference set *R*:
    - For each amino acid A:
      compute the relative frequencies of synonymous codons:

$$F^k(C_{A,j}) = \frac{q_{A,j}}{\sum_{j=1}^{m} q_{A,j}}, \sum_{j=1}^{m} F(C_{A,j}) = 1$$

      where $(C_{A,j})_{j=1}^{m}$ are *m* synonymous codons of said amino acid *A*; $q_{A,j}$ is the number of appearances of codon $C_{A,j}$ in the processed sequence; the superscript *k* stands for the k-th reference sequence.
    - For each amino acid, *A*, compute the average relative frequencies (or other translation rate parameter) of its synonymous codons over all sequences in *R*:

$$F(C_{A,j}) = \frac{1}{|R|} \sum_{k=1}^{|R|} F^k(C_{A,j})$$

- Initialize the re-engineered sequence s into the wild-type sequence *t*: s ← t
- For each codon $C_{A,\mathrm{i}}$ in *s*:

  Replace $C_{A,\mathrm{i}}$ with a synonymous codon $C_{A,\mathrm{j}}$ according to the synonymous rule **Q**, e.g.**:**

  $C_{A,j} = \underset{C_{A,k}}{\mathrm{argmax}}\, F_A(C_{A,k})$ for optimization or

  $C_{A,j} = \underset{C_{A,k}}{\mathrm{argmin}}\, F_A(C_{A,k})$ for de-optimization

- Estimate the optimization level ***L(s)*** using for example one of the following:

- **Euclidian distance** between two vectors representing relative synonymous codons frequencies of $s_1$ and $s_2$ (RSCF vectors); or
- **Mean typical decoding rate (MTDR)** as defined in sections 2.8-2.9
- **Relative translation elongation efficiency coefficient (RTEC).** Quantifies the relative differences in mean MTDR values of 2 groups of enfogenous viral genes.

$$\text{RTEC} = \frac{(mean\ MTDR_1 - mean\ MTDR_2)}{(mean\ MTDR_1 + mean\ MTDR_2)}$$

where E and L sigh for early and late genes. See also [2].

- **Codon Adaptation Index** (CAI) with respect to *R*. CAI is the most common technique for analyzing Codon usage bias. CAI measures the deviation of a given protein coding gene sequence with respect to a reference set of genes [3]. It is defined by

$$CAI = e^{\frac{1}{L}\sum_{l=1}^{L} \log (w_i(l))}$$

$$w_i = \frac{f_i}{\max (f_j)}$$

Where *w* is the weight of each codon calculated from a reference set, *f* is the codon frequency in the reference set genes, *i* and *j* are indexes of synonymous codons for amino acid and *L* is the gene length in codons unit.

- **Relative Codon De-optimization Index (RCDI)** with respect to *R* [4]:

$$\text{RCDI} = \frac{1}{\text{N}} \sum_{\text{i}} \frac{\text{F}_\text{s}(\text{C}_\text{i})}{\text{F}_\text{R}(\text{Ci})} \times \text{NC}_\text{i}$$

$\text{F}_\text{s}(\text{C}_\text{i})$ is the observed relative frequency in the tested sequence of each codon *i* out of all synonymous codons for the same amino acid (0 to 1), $\text{F}_\text{R}(\text{C}_\text{i})$is the relative frequency observed in the Reference set of each codon *i* out of all synonymous codons for that amino acid (0 to 1), $\text{NC}_\text{i}$ is the number of occurrences of that codon *i* in the sequence, and *N* is the total number of codons (amino acids) in the sequence. The higher the index, the more extensive deoptimization is.

## 2.2. Examples

### 2.2.1. Codon de-optimization of Human Simplexvirus (HHV-1) UL2 gene with respect to the set of viral early genes.

Viral genome was downloaded from www.ncbi.nlm.nih.gov/nuccore/NC_001806.2; genbank accession number NC_001806.2.

The deoptimized variant was engineered using the algorithm in section 2.1 with the following input/definitions:

- S = wildtype coding sequence of *UL2* gene.
- R $= (UL2, UL5, UL8, UL12, UL23, UL29, UL30, UL39, UL40, UL42, UL50, UL52, US3)$ – a reference set of simplexvirus early genes [5].
- The synonymous rule **Q** is defined as: substitute each wild type codon with the less frequent codon in R.
- 100 randomized variants were generated according to the average CUB of: (1) all viral genes; (2) human genes (http://www.kazusa.or.jp/codon/cgi-bin/showcodon.cgi?species=9606)
- The deoptimization evaluation function **L** was defined as: (1) Euclidean distance; (2) Codon Adaptation Index (CAI); (3) Relative Codon Deoptimization Index (RCDI)

Results:

Percentage of altered nucleotides in t with respect to s was found to be 37%.

The evaluation of the deoptimization levels in comparison to randomized variants is given in Figure S8:

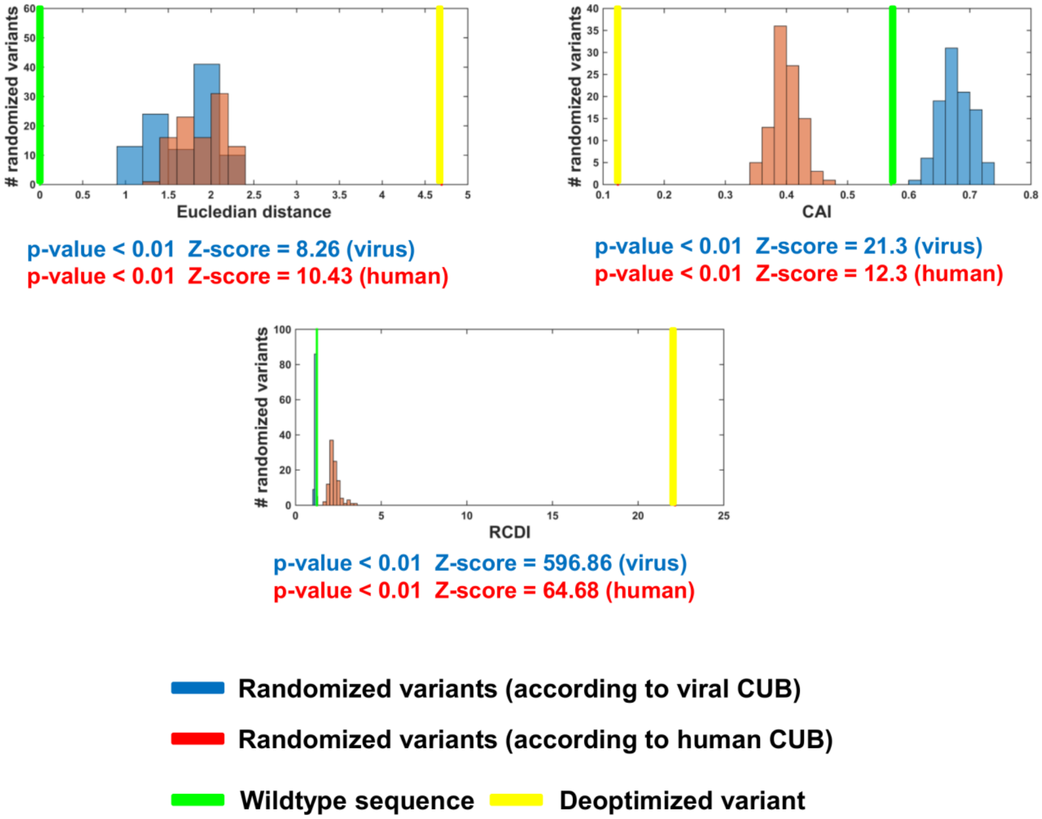


***Figure S8. Evaluation of Simplexvirus UL2 gene deoptimization using Eucledian distance(D), CAI and RCDI.*** *Deoptimized evaluation values:* ***D(s,t) = 4.69, CAI(t,R) = 0.12, RCDI(t,R) = 22.16*** *are given by yellow bars. Wildtype evaluation value:s* ***D(s,s) = 0, CAI(s,R) = 0.57, RCDI(s,R) = 1.24*** *are given by green bars.* *D(x,y) – Euclidian distance between synonymous codons relative frequency vectors of sequences x and y; CAI(x,R)/ RCDI(x,R) are CAI/RCDI values of sequence x with respect to the reference set R. The distribution of D/CAI/RCDI values for variants randomized with respect to the* average *viral CUB is given by blue bars; the distribution of D/CAI/RCDI values for variants randomized with respect to the average human CUB is given by red bars. P-value and Z-score estimations w.r.t each random model are specified.*

### 2.2.2. Codon optimization of human IL2 gene with respect to the set of Simplex virus early genes.

Viral genome was downloaded from www.ncbi.nlm.nih.gov/nuccore/NC_001806.2; genbank accession number NC_001806.2.

The coding sequence of human gene *IL2 (*interlukine 2*)* was downloaded from https://www.ncbi.nlm.nih.gov/nuccore/NM_000586; genbank accession number NM_000586.

The deoptimized variant was engineered using the algorithm in section 2.**1** with the following input/definitions:

- S = wildtype coding sequence of human gene *IL2*
- R = $(UL2, UL5, UL8, UL12, UL23, UL29, UL30, UL39, UL40, UL42, UL50, UL52, US3)$ – a reference set of Simplexvirus early genes [5].
- The synonymous rule **Q** is defined as: substitute each wild type codon with the most frequent codon in R.
- 100 randomized variants were generated according to the average CUB of: (1) all viral genes; (2) human genes (http://www.kazusa.or.jp/codon/cgi-bin/showcodon.cgi?species=9606)
- The deoptimization evaluation function **L** was defined as: (1) Euclidean distance; (2) Codon Adaptation Index (CAI)

Results:

Percentage of altered nucleotides in t with respect to s = 25%

The evaluation of the optimization levels in comparison to randomized variants is given in figure S9:

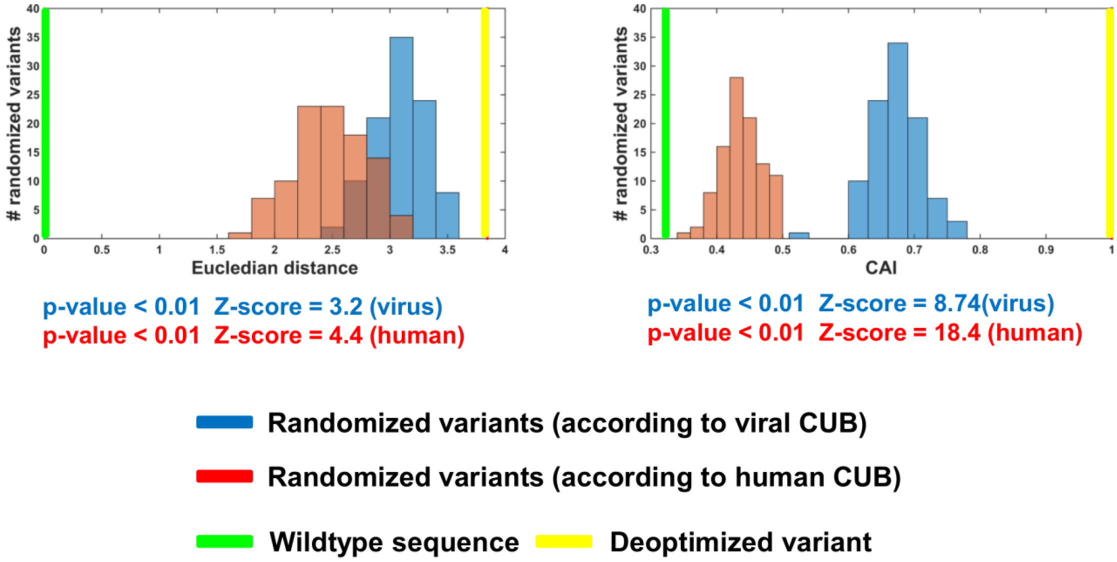


**Figure S9.** ***Evaluation of human IL2 gene optimization using Eucledian distance(D) and CAI.*** *Optimized evaluation values* ***D(s,t) = 3.84, CAI(t,R) = 1*** *are given by yellow bars. Wildtype evaluation values* ***D(s,s) = 0, CAI(s,R) = 0.32*** *are given by green bars.* *D(x,y) – Euclidian distance between synonymous codons relative frequency vectors of sequences x and y; CAI(x,R) are CAI values of sequence x with respect to the reference set R. The distribution of D/CAI values for variants randomized with respect to the average viral CUB is given by blue bars; the distribution of D/CAI values for variants randomized with respect to the average human CUB is given by red bars. P-value and z-score estimations w.r.t each random model are specified*

## 3. Methods

### 3.1. Viruses

We analyzed viruses with a known division to early and late genes based on the literature. The list of sources for early and late genes appear in Table S3 below.

**Table S3. List of sources of late and early genes.**

| **Virus** | **References** |
|---|---|
| **Phages** | |
| **T4** | • Luke, K., Radek, A., Liu, X., Campbell, J., Uzan, M., Haselkorn, R., & Kogan, Y. (2002). Microarray analysis of gene expression |

| | |
|---|---|
| | during bacteriophage T4 infection. Virology, 299(2), 182-191. |
| **Pak_P3** | • Chevallereau, A., Blasdel, B. G., De Smet, J., Monot, M., Zimmermann, M., Kogadeeva, M., ... & Lavigne, R. (2016). Next-generation "-omics" approaches reveal a massive alteration of host RNA metabolism during bacteriophage infection of Pseudomonas aeruginosa. PLoS genetics, 12(7), e1006134. |
| **phi29** | • Hirofumi, Y., & Ito, J. (1982). Nucleotide sequence of the major early region of bacteriophage φ29. Gene, 17(3), 323-335.<br>• http://viralzone.expasy.org/ |
| **T7** | • Dunn, J. J., Studier, F. W., & Gottesman, M. (1983). Complete nucleotide sequence of bacteriophage T7 DNA and the locations of T7 genetic elements. Journal of molecular biology, 166(4), 477-535. |
| **phiYs40** | • Sevostyanova, A., Djordjevic, M., Kuznedelov, K., Naryshkina, T., Gelfand, M. S., Severinov, K., & Minakhin, L. (2007). Temporal regulation of viral transcription during development of Thermus thermophilus bacteriophage ϕYS40. Journal of molecular biology, 366(2), 420-435. |
| **Fah** | • Minakhin, L., Semenova, E., Liu, J., Vasilov, A., Severinova, E., Gabisonia, T., ... & Severinov, K. (2005). Genome sequence and gene expression of Bacillus anthracis bacteriophage Fah. Journal of molecular biology, 354(1), 1-15. |
| **xp10** | • Semenova, E., Nagornykh, M., Pyatnitskiy, M., Artamonova, I. I., & Severinov, K. (2009). Analysis of CRISPR system function in plant pathogen Xanthomonas oryzae. FEMS microbiology letters, 296(1), 110-116. |
| **Streptococcus DT1** | • Duplessis, M., Russell, W. M., Romero, D. A., & Moineau, S. (2005). Global gene expression analysis of two Streptococcus thermophilus bacteriophages using DNA microarray. Virology, 340(2), 192-208. |

| | |
|---|---|
| **Streptococcus 2972** | • Duplessis, M., Russell, W. M., Romero, D. A., & Moineau, S. (2005). Global gene expression analysis of two Streptococcus thermophilus bacteriophages using DNA microarray. Virology, 340(2), 192-208. |
| **Mu** | • http://viralzone.expasy.org/ |
| **phiC31** | • Smith, M. C., Burns, R. N., Wilson, S. E., & Gregory, M. A. (1999). The complete genome sequence of the Streptomyces temperate phage φC31: evolutionary relationships to other viruses. Nucleic acids research, 27(10), 2145-2155. |
| **phiEco32** | • Pavlova, O., Lavysh, D., Klimuk, E., Djordjevic, M., Ravcheev, D. A., Gelfand, M. S., ... & Akulenko, N. (2012). Temporal regulation of gene expression of the Escherichia coli bacteriophage phiEco32. Journal of molecular biology, 416(3), 389-399. |
| **p23-45** | • Berdygulova, Z., Westblade, L. F., Florens, L., Koonin, E. V., Chait, B. T., Ramanculov, E., ... & Minakhin, L. (2011). Temporal regulation of gene expression of the Thermus thermophilus bacteriophage P23-45. Journal of molecular biology, 405(1), 125-142. |
| **phiR1-37** | • Leskinen, K., Blasdel, B. G., Lavigne, R., & Skurnik, M. (2016). RNA-sequencing reveals the progression of phage-host interactions between φR1-37 and Yersinia enterocolitica. Viruses, 8(4), 111. |
| **Human viruses** | |
| **Simplex virus** | • Roizman, B. (1996). The function of herpes simplex virus genes: a primer for genetic engineering of novel vectors. Proceedings of the National Academy of Sciences, 93(21), 11307-11312.<br>• http://www.uniprot.org/ |
| **Varicello virus** | • Liu, X., Li, Q., Dowdell, K., Fischer, E. R., & Cohen, J. I. (2012). Varicella-zoster virus ORF12 protein triggers |

| | |
|---|---|
| | phosphorylation of ERK1/2 and inhibits apoptosis. Journal of virology, 86(6), 3143-3151.<br>• http://www.uniprot.org/<br>• Cohen, J. I., Brunell, P. A., Straus, S. E., & Krause, P. R. (1999). Recent advances in varicella-zoster virus infection. Annals of Internal Medicine, 130(11), 922-932. |
| **Cytomegalo virus** | • Hengel, H., Brune, W., & Koszinowski, U. H. (1998). Immune evasion by cytomegalovirus—survival strategies of a highly adapted opportunist. Trends in microbiology, 6(5), 190-197.<br>• http://www.uniprot.org/<br>• Herpes homologs table* |
| **Roseolo virus** | • Herpes homologs table*<br>• http://www.uniprot.org/<br>• https://en.wikipedia.org/wiki/Human_herpesvirus_6#Genes |
| **Alphapapilloma virus** | • http://viralzone.expasy.org/ |
| **Betapapilloma virus** | • http://viralzone.expasy.org/ |
| **Gammapapilloma virus** | • http://viralzone.expasy.org/ |
| **Mupapilloma virus** | • http://viralzone.expasy.org/ |
| **Nupapilloma virus** | • http://viralzone.expasy.org/ |
| **Polyoma virus** | • http://viralzone.expasy.org/ |
| **HIV-1** | • Cullen, BRYAN R. "Regulation of HIV-1 gene expression." The FASEB Journal 5.10 (1991): 2361-2368. |

*Herpes homologs table was taken from:

Nicholas, J. (1996). Determination and analysis of the complete nucleotide sequence of human herpesvirus. Journal of virology, 70(9), 5975-5989.

### 3.2. The mechanism of regulation of early/late genes in HIV

According to the study 'Regulation of HIV-1 gene expression' [6], the mRNA species encoded by HIV-1 can be divided into two classes based on their temporal expression during the HIV-1 replication cycle. The early class of viral mRNAs consists of the multiply spliced, -2-kb mRNA species that encode the viral regulatory proteins Tat, Nef, and Rev. The late class of viral mRNAs consists of the unspliced (-9 kb) and singly spliced (-4 kb) transcripts that encode the virion structural proteins. Rev mutants of HIV-1 are incapable of inducing synthesis of the viral structural proteins, and are therefore replication defective. The switch from the early, regulatory phase of HIV-1 gene expression to the late, structural phase appears to require expression of a critical level of the Rev protein. The primary role of the Rev regulatory pathway may therefore be to prevent premature progression of the viral replication cycle to the late or lytic phase in cells which are incapable of supporting a sufficient level of viral mRNA and protein synthesis. Tat activate gene expression and can lead to high protein synthesis (positive feedback). In addition to Tat and Rev, HIV-1 encodes four other auxiliary proteins named Nef, Vpr, Vif, and Vpu. Nef, the third early gene product of HIV-1, is a myristylated phosphoprotein that is associated with cytoplasmic membrane structures. Unlike Tat and Rev, the Nef gene product is not required for HIV-1 replication in culture. The role of the Nef gene product remains unclear, likely to play a significant role in the viral life cycle in the infected host. The HIV-1 Vif, Vpu, and Vpr gene products are cytoplasmic proteins that are expressed late in the HIV-1 replication cycle in a Rev-dependent manner. The role played by these proteins is primarily structural rather than regulatory. Vif and Vpu have been shown to function in the morphogenesis and release of infectious HIV-1 virions while the Vpr protein appears to be virion-associated. All three of these proteins significantly enhance replication of HIV-1 in culture.

### 3.3. Synonymous codon usage analysis

Codon composition of a coding sequence was represented by a 61-dimensional vector of relative synonymous codons frequencies (RSCF) of each one of 61 coding codons (stop codons are excluded):

$$RSCF = (RSCF[1], \dots, RSCF[61])$$

$$RSCF[i] = \frac{q_i}{\sum_{j \in syn[i]} q_j}, \sum_{j \in syn[i]} RSCF[j] = 1$$

where $q_i$ is the number of appearances of codon $i$ in a sequence, *syn[i]* is a subset of indexes in RSCF pointing at codons synonymous to codon $i$.

Clustering analysis was performed on RSCF vectors of each viral coding sequence. In order to exclude biases due to a possible absence of specific amino acids in specific sequences (missing amino acids), the relative synonymous frequency of a codon corresponding to a missing amino acid was set to the average relative synonymous frequency of this codon over all sequences in which at least one such amino acid is present.

Each viral sequence was assigned a group label corresponding to its temporal expression stage (Early/Late) (according to the classification known in the literature). The tendency of sequences to cluster according to the codons usage in two different clusters corresponding to their temporal expression stages (early/late) was measured using the Davies-Bouldin score (DBS). This score is based on a ratio of within-cluster and between-cluster distances and is defined as:

$$DBS = \frac{1}{k}\sum_{i=1}^{k} \max_{j \neq i}\{D_{ij}\}, \qquad D_{ij} = \frac{\overline{s_i} + \overline{s_j}}{M_{ij}}$$

Where k – is the number of evaluated clusters, $D_{ij}$ is the within-to-between cluster distance ratio for the i-th and j-th clusters; $\overline{s_i}$ is the standard deviation of the Euclidian distance between each point in the i-th cluster and the centroid of the i-th cluster; $\overline{s_j}$ is the standard deviation of the Euclidian distance between each point in the j-th cluster and the centroid of the j-th cluster; $M_{ij}$ is the Euclidean distance between the centroids of the ith and jth clusters. The maximum value of $D_{ij}$ represents the worst-case within-to-between cluster ratio for cluster i. The optimal clustering solution has the smallest Davies-Bouldin score value.

The significance of cluster separation was assessed by comparing the DBS of the wildtype sequences to the randomized scores obtained from 1000 permutations of gene group labels (early or late).

In order to visualize the clustering, a principal component analysis (PCA) was applied to project the RSCF vectors to a plane spanned by their first two principal components. In order to

visualize the separation between clusters a maximum margin separation line - a line for which the distance between it and the nearest point from either of the groups is maximized, was calculated and plotted.

In addition, the above cluster analysis was performed on amino acid frequencies as well.

### 3.4. The tRNA adaptation index (tAI)

tAI quantifies the adaptation of a coding region to the tRNA pool. Let tCGNi j be the copy number of the j-th anti-codon that recognizes the i-th codon and let Si j be the selective constraint of the codon–anti-codon coupling efficiency. Thus, the absolute adaptiveness value of a codon of type i (1 ≤ i ≤ 61; stop codons are excluded) to the tRNA pool is defined by:

$$\mathrm{W_i} = \sum_{\mathrm{j=1}}^{\mathrm{n_i}} \left(1 - \mathrm{S_{ij}}\right)\mathrm{tCGN_{ij}}$$

For each amino acid, the weight of each of its codons, is computed as the ratio between the absolute adaptiveness value of the codon and the maximal absolute adaptiveness value of the synonymous codons for that amino acid:

$$w_i = \frac{W_i}{\max\limits_{j \in syn[i]} W_j}$$

where $W_i$ is the absolute adaptiveness of codon *i* in a sequence, *syn[i]* is a subset of indexes in pointing at codons synonymous to codon *i*. $w_i$ takes values from 0 (not adapted) to 1(maximally adapted). If the weight value is zero a value of 0.5 is used

tAI is the geometric mean of $w_i$ (relative codon-tRNA adaptation) over codons of a coding sequence.

Although the tRNA adaptation index (tAI) is a widely used measure of the efficiency by which a coding sequence is recognized by the intra-cellular tRNA pool, currently, the codon-anticodon efficiency weights are based on the gene expression measurments of only a limited number of organisms. However, the efficiencies of the different codon-tRNA interactions are expected to vary among different organisms and cannot be interchangeably used in different species. In [7, 8] a new approach for adjusting the tAI weights to any target model organism without the need for

gene expression measurements, basing on optimizing the correlation between the tAI and a measure of codon usage bias was developed and implemented. Here we apply this approach for the first time for estimating tAI in viruses with respect to their hosts.

### 3.5. Effective number of codons (ENC)

ENC is a measure that quantifies how far the codon usage of a gene departs from equal usage of synonymous codons [9]. It can be calculated from codon usage data alone, and is independent of gene length and amino acid (AA) composition. ENC can take values from 20, in the case of extreme bias where one codon is exclusively used for each aa, to 61 when the use of alternative synonymous codons is equally likely and is defined by:

$$n = \sum_{i}^{d} x_i$$

$$p_i = x_i / n$$

xi is the number of synonymous codons of each type in the sequence, n is the number of times the AA appears in the sequence and p is the frequency (/probability) of each codon.

The effective number of codons for the AA is:

$$\widehat{N_e} = 1/\hat{F} \text{ where } \hat{F} = \sum_i^d p_i^2$$

ENC for the group of AA with degeneracy *d*:

$$N = 1/\overline{\widehat{F_d}} \text{ where } \overline{\widehat{F_d}} = \frac{1}{|A_d|} \sum_{i \in A_d} \widehat{F_i}$$

When an AA is missing we averaged over the rest.

Finally, ENC for the sequence (e.g., gene):

$$\widehat{N_e} = 2 + \frac{9}{\overline{\widehat{F_2}}} + \frac{1}{\overline{\widehat{F_3}}} + \frac{5}{\overline{\widehat{F_4}}} + \frac{3}{\overline{\widehat{F_6}}}$$

### 3.6. GC content

GC-content (or guanine-cytosine content) is the percentage of nitrogenous bases on a DNA or RNA molecule that are either guanine or cytosine (from a possibility of four different nucleotides) and is defined as:

$$\frac{F(G)+F(C)}{F(A)+F(T)+F(G)+F(C)}$$

Where F() is a number of occurrences. We calculated the GC content in gene levels for all viruses.

### 3.7. Codon pair bias (CPB)

To quantify codon pair bias, we follow [10] and define a codon pair score (CPS) as the log ratio of the observed over the expected number of occurrences of this codon pair in the coding sequence. To achieve independence from amino acid and codon bias, the expected frequency is calculated based on the relative proportion of the number of times an amino acid is encoded by a specific codon:

$$CPS = \log\left(\frac{F(AB)}{\frac{F(A)\times F(B)}{F(X)\times F(Y)}\times F(XY)}\right),$$

where the codon pair AB encodes for amino acid pair XY and F denotes the number of occurrences. The codon pair bias (CPB) of a virus is than defined as an avenge codon pair scores over all codon pairs comprising all viral coding sequences:

$$CPB = \frac{1}{k-1}\sum_{i=1}^{k-1} CPS[i]$$

### 3.8. Dinucleotide bias (DNTB)

Following [11] we compute a dinucleotide score (DNTS) for a pair of nucleotides XY as an odds ratio:

$$DNTS = \frac{F(XY)}{F(X)F(Y)},$$

where F denotes the frequency of occurrences. The dinucleotide pair bias (DNTB) of a virus is defined as an average of dinucleotide scores over all dinucleotides comprising all viral sequences:

$$DNTB = \frac{1}{k-1}\sum_{i=1}^{k-1} DNTS[i]$$

### 3.9. Nucleotide bias (NTB) and Amino acid bias (AAB)

Nucleotide bias and amino acid bias are calculated as normalized Shannon entropy over the frequencies of the nucleotides/ amino acids in the sequence (gene or genome) and is defined as:

$$E = -\frac{\sum_i F * log_2(F)}{|unique\ symbols|}$$

Where E is entropy, F is the frequency of nucleotides or amino acids and the number of unique symbols equals to 4 in the case of nucleotides or 20 in the case of amino acids. This measure takes values between 0 and 1, and describes how dispersed the distribution of the alphabet elements is: higher values correspond to more uniform nucleotide usage; lower values correspond to more biased nucleotide, indicating that some nucleotides/amino acids are preferred (positions are conserved)

### 3.10. Codon typical decoding rate (TDR)

Ribosome profiles for bacteriophage Lambda expressed genes were reconstructed and normalized. The normalization enables measuring the relative time a ribosome spends translating each codon in a specific gene relative to other codons in it, while considering the total number of codons in the gene, resulting in its normalized footprint count (NFC):

$$\mathrm{NFC_j} = \frac{\mathrm{RC_j}}{\frac{1}{\mathrm{J}-40}\left(\mathrm{RC_{21}} + \mathrm{RC_{22}} + \cdots + \mathrm{RC_{J-20}}\right)}$$

$$j = 21 \ldots J - 20$$

Where RC stands for read count; J is the number of codons in the gene, and j is the index of a codon. A histogram of NFC values for each codon was generated. Each NFC distribution describes the probability of observing each of the codon's NFC values in the ORFs of the analyzed organism.

To estimate the typical decoding time of each codon based on NFC distributions, we used a novel statistical model [12] which takes into consideration the skewed nature of the NFC distribution. The aim is to describe the NFC histogram of each codon as an output of a random variable which is a sum of two random variables: a normal and an exponential variable. Thus, the distribution of this new random variable includes three parameters, and is called EMG distribution. In this model, the typical codon decoding time was described by the normal distribution with two parameters: mean (μ) and standard deviation (σ); the μ parameter represents the location of the mean of the theoretical Gaussian component that should be obtained if there are no phenomena such as pauses/ biases/ ribosomal traffic jams; σ represents the width of the Gaussian component. The exponential distribution has one parameter λ, which represents the skewedness of the NFC distribution due to reasons such as ribosomal jamming caused by codons with different decoding times, extreme pauses, incomplete halting of the ribosomes, biases in the experiment, etc. The EMG is defined as follows:

$$f(x;\mu,\sigma,\lambda) = \frac{\lambda}{2} e^{\frac{\lambda}{2}(2\mu+\lambda\sigma^2-2x)} \operatorname{erfc}\left(\frac{\mu+\lambda\sigma^2-x}{\sqrt{2}\sigma}\right),$$

$$\operatorname{erfc}(x) = 1 - \operatorname{erf}(x) = \int_x^{\infty} e^{-t^2} dt$$

Maximum likelihood criterion was used to estimate these three parameters for each codon based on the ribosome profiling data by fitting the suggested model to the NFC distribution. $\frac{1}{\mu}$ was defined to be the Typical Decoding Rate (TDR) of each codon.

In order to optimize the TDR according to the actual riboseq read counts in every time condition; outliers were removed from the NFC distribution of each codon in the following way: for every codon (in every time condition), and for each NFCi point related to the codon, we calculated the probability (Pi) to see value larger or equal to NFCi based on the pdf fitted to the codon (EMG

distribution). Let Ni denote the number of measurements of the codon NFC based on the data; points in which the result of $\mathrm{p_i} * \mathrm{N_i}$ was lower than 0.001 were removed. The NFC and TDR values were calculated as in our previous study [2].

### 3.11. Mean typical decoding rate (MTDR).

A measure which estimates the global translation elongation efficiency of the entire gene as a geometric average of typical decoding rates of its codons:

$$\mathrm{MTDR} = \mathrm{e}^{\frac{1}{\mathrm{L}}\sum_{\mathrm{l}=1}^{\mathrm{L}} \log\left(\mathrm{TDR_i(l)}\right)}$$

where i is an index of a codon and L is the gene length in codon unit.

### 3.12. Phylogenetic Reconstruction.

Let A and B be two proteomes of two different viruses consisting of n and m proteins correspondingly. The total length of each proteomes is: $|A| = \sum_{i=1}^{n} |a_i|\ and\ |B| = \sum_{i=1}^{m} |b_i|$ (|x| is the length of protein x). We define an ARS score of a protein $a_i \in A$ with respect to the proteome B as:

$$L(a_i, B) = \frac{1}{a_i} \sum_{j=1}^{|a_i|} l_i(j)$$

Where $l_i(j)$ is the length of the longest substring$[a_i(j)a_i(j+1) \ldots a_i(j + l(j) - 1)]$, starting at position j in $a_i$ that exactly matches a substring $[b_\gamma(k)b_\gamma(k+1) \ldots b_\gamma(k + l(j) - 1)]$ starting at some position k in one of the proteins $b_\gamma \in B$.

The ARS score of the entire proteome A with respect to the proteome B is defined as:

$$L(A, B) = \frac{1}{|A|} \sum_{i=1}^{n} [|a_i| L(a_i, B)] = \frac{1}{|A|} \sum_{i=1}^{n} \sum_{j=1}^{|a_i|} l_i(j)$$

The first equality is a weighted average of ARS scores $L(a_i, B)$ over all proteins in A; for each protein, $a_i \in A$ its weight is defined as its relative lengths $\frac{|a_i|}{|A|}$. The second equality means that the weighted average described above is just equal to the total sum of the longest common

substrings $l_i(j)$ over all positions j in all proteins $a_i$, divided by the total length of the proteome A (this equivalence immediately follows from the definition of $L(a_i, B)$ ) .

Intuitively, the larger this L(A,B) is, the more similar the two proteomes are.

For a given A, to account for B's length (longer B will tend to have larger L(A,B) we normalize by log(|B|): L(A,B) / log(|B|).

Moreover, L(A,B) is a similarity measure, while we are after distance d. Therefore, we take the inverse and then subtract a "correction term" that guarantees d(A, A) will always be zero, yielding:

$$d(A,B) = \frac{\log(|B|)}{L(A,B)} - \frac{\log(|A|)}{L(A,A)}$$

Note that if A is a single protein, then by the definition of ARS and a formula for the sum of geometric progression:

$$L(A,A) = \frac{1}{n}\sum_{j=1}^{n} l(i) = \frac{1}{n}\sum_{j=1}^{n}(n-i+1) = \frac{1}{2n} * n(n+1) = \frac{n+1}{2}$$

However, if A is a proteome that contains several proteins than this formula is no more applicable; in this case L(A,A) should be computed by the formula of L(A,B).

Finally, to ensure symmetricity we define:

$$d_s = \frac{d(A,B) + d(B,A)}{2}$$

ds is the ARS induced distance for tree building.

The tree was built using neighbor joining algorithm.

## 4. References


1. Ulitsky I, Burstein D, Tuller T, Chor B: **The average common substring approach to phylogenomic reconstruction**. *Journal of Computational Biology* 2006, **13**(2):336-350.
2. Goz E, Mioduser, O., Diament, A. and Tuller, T.: **Evidence of translation efficiency adaptation of the coding regions of the bacteriophage lambda**. *DNA Research* 2017, **24**(4):333-342.
3. Sharp PM, Li WH: **THE CODON ADAPTATION INDEX - A MEASURE OF DIRECTIONAL SYNONYMOUS CODON USAGE BIAS, AND ITS POTENTIAL APPLICATIONS**. *Nucleic Acids Research* 1987, **15**(3):1281-1295.

4. Mueller S, Papamichail D, Coleman JR, Skiena S, Wimmer E: **Reduction of the rate of poliovirus protein synthesis through large-scale codon deoptimization causes attenuation of viral virulence by lowering specific infectivity**. *Journal of Virology* 2006, **80**(19):9687-9696.
5. Roizman B: **The function of herpes simplex virus genes: A primer for genetic engineering of novel vectors**. *Proceedings of the National Academy of Sciences of the United States of America* 1996, **93**(21):11307-11312.
6. Cullen BR: **Regulation of HIV-1 gene expression**. *The FASEB Journal* 1991, **5**(10):2361-2368.
7. Sabi R, Daniel RV, Tuller T: **stAI(calc): tRNA adaptation index calculator based on species-specific weights**. *Bioinformatics* 2017, **33**(4):589-591.
8. Sabi R, Tuller T: **Modelling the Efficiency of Codon-tRNA Interactions Based on Codon Usage Bias**. *DNA Research* 2014, **21**(5):511-525.
9. Wright F: **THE EFFECTIVE NUMBER OF CODONS USED IN A GENE**. *Gene* 1990, **87**(1):23-29.
10. Coleman JR, Papamichail D, Skiena S, Futcher B, Wimmer E, Mueller S: **Virus attenuation by genome-scale changes in codon pair bias**. *Science* 2008, **320**(5884):1784-1787.
11. Karlin S: **Global dinucleotide signatures and analysis of genomic heterogeneity**. *Current Opinion in Microbiology* 1998, **1**(5):598-610.
12. Dana A, Tuller T: **The effect of tRNA levels on decoding times of mRNA codons**. *Nucleic Acids Research* 2014, **42**(14):9171-9181.
